# Analysis of regional differences in the amount of hypnotic and anxiolytic prescriptions in Japan using nationwide claims data

**DOI:** 10.1186/s12888-021-03657-6

**Published:** 2022-01-19

**Authors:** Tasuku Okui, Jinsang Park

**Affiliations:** 1 Medical Information Center, Kyushu University Hospital, 812-8582 Maidashi3-1-1 Higashi-ku, Fukuoka City, Fukuoka Prefecture Japan; 2 Department of Pharmaceutical Sciences, International University of Health and Welfare, 831-8501 Enokizu 137-1, Okawa, Fukuoka Prefecture Japan

**Keywords:** Hypnotics, Anxiolytics, Obesity, Prescriptions, Japan, Socioeconomic factors

## Abstract

**Background:**

In Japan, there has been no investigation of regional differences in the number or amount of prescriptions of anxiolytics or hypnotics. Attributes related to the high amount of prescriptions for these drugs are unknown. We investigated recent trends and regional differences in the amount of prescriptions of hypnotics and anxiolytics in Japan and identified factors associated with these regional differences.

**Methods:**

The National Database of Health Insurance Claims and Specific Health Checkups of Japan (NDB) Open data from 2015 to 2018 were used. We calculated diazepam-equivalent doses (mg) for each drug and the total amount of prescriptions per capita for hypnotics and anxiolytics by sex and age. In addition, we calculated the standardized claim ratio (SCR) of the amount of prescriptions by prefecture. We investigated factors associated with regional differences in the SCRs of hypnotics and anxiolytics using the prefectures’ medical, socioeconomic, and physical characteristics by an ecological study using a linear mixed-effects model.

**Results:**

The amount of prescriptions of hypnotics and anxiolytics, specifically, the amount of prescriptions of benzodiazepine receptor agonists (BZRAs), decreased in many of the adult age groups from 2015 to 2018. The regression analysis revealed that the number of medical clinics per capita, the number of public assistance recipients per capita, the proportion of persons whose HbA1c ≥ 6.5%, and the proportion of persons whose BMI ≥25 kg/m^2^ were positively and significantly associated with the SCR of hypnosis. In contrast, the number of public assistance recipients per capita and the proportion of persons whose BMI ≥25 kg/m^2^ were positively and significantly associated with the SCR of anxiolytics.

**Conclusions:**

Factors associated with prescription amount of hypnotics and anxiolytics were revealed in this study, and a further study is needed for investigating causal relationships between the prescriptions amount and the associated factors using individual data.

## Background

Multidrug prescriptions or polypharmacy of psychotropic drugs is a significant public health problem in Japan. In recent years, there were some published guidelines and revisions of medical fees. In 2012, prescription fees that a hospital can receive from the “Healthcare bill check and payment organization” began to decrease when three or more types of hypnotics or anxiolytics were prescribed simultaneously [1]. After that, restrictions of multidrug prescription increased every time a medical fee revision was conducted. Prescription fees began to be reduced from 2018 also when four or more types of drugs (hypnotics or anxiolytics) were prescribed simultaneously [1]. Particularly, the number of benzodiazepine receptor agonists (BZRAs) prescriptions is high among hypnotics and anxiolytics in Japan [2]. Also, many studies reported adverse effects of BZRAs, such as falls and venous thromboembolism [3, 4]. However, in recent years, hypnotics and anxiolytics or BZRAs prescription trends have not been revealed using nationwide statistics data. It is important to understand the attributes of patients who receive large numbers of prescriptions and reduce psychotropic drug use.

Some studies investigated prescription trends or the amount of anxiolytics or hypnotics prescribed in Japan [5, 6]. However, few studies investigated the physical or socioeconomic attributes of patients prescribed anxiolytics or hypnotics [7]. Also, few studies investigated regional differences in the prevalence of patients with psychiatric diseases or the amount of prescriptions of psychotropic drugs [8]. No studies investigated regional differences in the number of prescriptions of anxiolytics or hypnotics. By revealing regional differences in the amount of prescriptions of anxiolytics or hypnotics, and finding factors associated with the difference, we can infer the physical or socioeconomic attributes related to the prescription. We can also identify prefectures with high amount of prescriptions, and we might be able to inspect the effect of the high number of prescriptions on residents’ health or take some administrative measures against those prefectures. The National Database of Health Insurance Claims and Specific Health Checkups of Japan (NDB) has valuable data for revealing the actual conditions of prescriptions in Japan [9]. The NDB is an accumulation of health insurance claims data from all of Japan. These data have been used for revealing the prescription trends of some drugs [8, 10].

This study aimed to determine the trend and regional differences in the amount of prescriptions of hypnotics and anxiolytics in Japan using NDB data and identify the factors associated with the regional differences by an ecological study.

## Methods

We used NDB Open data from 2015 to 2018 in this analysis [11]. Aggregated data of the NDB are publicly available as NDB Open data, and data on the amount of prescriptions of drugs in all of Japan are also available. We used data on the amount of prescriptions of internal medicines by age groups and sex and data on the amount of prescriptions by medicines and prefectures. Data on the amount of in-hospital, out-of-hospital, and hospitalization prescriptions are available, and we used all the data. We used data on internal medicines whose generic names are designated as hypnotics and anxiolytics by the Ministry of Health, Labor, and Welfare [12]. Although phenobarbital is included as a hypnotics [12], we did not include it in the analysis because it is mainly used as an antiepileptic drug. The drug unit is different depending on the drug, and there were units, such as a tablet, ml, g, capsule, and mg. Therefore, we converted the units of all the prescription data into mg by surveying the contents of each drug. In addition, a titer (force of drug) differs depending on the generic name of the drug [13]. Therefore, we calculated diazepam-equivalent doses (mg) of the amount of prescriptions for each drug as in a previous study [5]. However, titers of suvorexant, triclofos sodium, hydroxyzine pamoate, ramelteon, gamma oryzanol, and hydroxyzine hydrochloride were unknown. Thus, we used the mean titers of other hypnotics and anziolytics as the titers of these drugs. In addition, we obtained population data by sex and age group from data on the survey of the basic resident register in Japan [14]. In addition, data of drugs whose prescription amount was not available in all age groups or all prefectures were removed from the analysis.

We calculated the amount of prescriptions (mg) by sex, age group, and year per person for hypnotics, anxiolytics, BZRAs, and Z-drugs. In addition, we calculated the amount of prescriptions per person for each prefecture by year. Age group and sex-specific prescription data are not available for each prefecture, and we calculated the standardized claim ratios (SCRs) for the amount of prescriptions of hypnotics and anxiolytics to correct differences in age and sex distribution among prefectures. We used the same SCR calculation method as in previous studies [15, 16], and the mean of the SCR is 100.

In addition, we investigated factors associated with regional differences in the SCRs of hypnotics and anxiolytics using the prefectures’ medical, socioeconomic, and physical characteristics. As medical characteristics, we used the number of medical clinics per 100,000 persons, the number of hospitals per 100,000 persons, the number of pharmacies per 100,000 persons, the number of hospitals with a psychiatric department per 100,000 persons, and the number of psychiatric beds per 100,000 persons. These variables were obtained from the Survey of medical institutions and the Report on Public Health Administration and Services [17]. We used the number of public assistance recipients per 1000 persons and taxable income per capita regarding socioeconomic characteristics. We obtained data on the number of public assistance recipients from the National Survey on Public Assistance Recipients [17]. Data on taxable income were obtained from the Survey of Status of Taxation for Municipal Tax [17].

Regarding physical characteristics of prefectures, the proportion of persons with an HbA1c ≥ 6.5%, the proportion of persons whose systolic blood pressure (SBP) ≥ 140 mmHg, the proportion of persons whose body mass index (BMI) ≥ 25 kg/m^2^, and the proportion of persons whose triglycerides ≥150 mg/dl were used. Persons with these characteristics are diagnosed with diabetes, hypertension, obesity, and dyslipidemia, respectively, by Japanese criteria. The data on these variables came from the Specific Health Checkups [11], which target all the insured persons aged 40–74 years in Japan [18].

We used a linear mixed-effects model to investigate the association between the SCR and the explanatory variables and conducted an ecological study using prefecture data. We used each prefecture as a random effect in the analysis, and adopted a random intercept and slope model. We calculated a standardized partial regression coefficient (SPRC) and 95% confidence intervals for each explanatory variable. All the statistical analyses were conducted using R version 3.6.3 [19].

## Results

Table 1 shows the number of drugs for hypnotics and anxiolytics available in the NDB Open data by each generic name of drug and year. BZRAs account for most hypnotics and anxiolytics. The yearly fluctuation in the number of drugs in the data for each generic name was relatively small.

**Table 1:** Number of types of drugs by generic name of drug and year in the data

					Year			
Typr of drug	Hypnotics	Anxiolytics	BZRAs	Z-drugs	2015	2016	2017	2018
Estazolam	〇		〇		9	9	9	10
Flurazepam		〇	〇		1	1	1	1
Nitrazepam	〇		〇		16	14	14	14
Nimetazepam	〇		〇		1	0	0	0
Triazolam	〇		〇		19	17	17	17
Flunitrazepam	〇		〇		21	22	22	24
Brotizolam	〇		〇		40	43	45	41
Lormetazepam	〇		〇		5	5	4	3
Oxazolam		〇	〇		2	2	2	2
Cloxazolam		〇	〇		6	6	6	6
Diazepam		〇	〇		25	24	24	23
Fludiazepam		〇	〇		2	2	2	2
Bromazepam		〇	〇		13	13	13	13
Medazepam		〇	〇		2	1	1	0
Lorazepam		〇	〇		12	12	12	12
Alprazolam		〇	〇		18	15	15	15
Flutazolam		〇	〇		1	0	0	0
Tofisopam		〇	〇		6	9	8	7
Flutoprazepam		〇	〇		1	0	0	0
Ethyl loflazepate		〇	〇		8	9	9	9
Quazepam	〇		〇		4	3	3	2
Triclofos sodium	〇				3	3	3	3
Rilmazafone	〇		〇		10	10	10	10
Zopiclone	〇		〇	〇	17	17	15	15
Tandospirone citrate		〇			5	3	3	4
Zolpidem	〇		〇	〇	39	46	49	54
Eszopiclone	〇		〇	〇	7	7	7	8
Hydroxyzine hydrochloride		〇			2	2	2	2
Clotiazepam		〇	〇		10	10	10	10
Hydroxyzine pamoate		〇			3	3	3	3
Etizolam		〇	〇		38	37	37	39
Ramelteon	〇				3	3	3	3
Suvorexant	〇				6	6	9	9
Gamma Oryzanol		〇			32	29	30	26

*BZRAs* benzodiazepine receptor agonists

Table 2 shows the prescription amount (Diazepam-equivalent doses in milligrams) per capita by type of drugs for men. BZRAs comprise most of the amount of prescriptions of hypnotics and anxiolytics. The amount of prescriptions decreased in many age groups except for younger age groups. This decrease did not occur in Z-drugs in young and middle-aged persons, although a decrease was observed among older age groups.

**Table 2:** Prescription amount (Diazepam-equivalent doses in milligrams) per capita by type of drugs for men

	Age group																		
Year	0–4	5–9	10–14	15–19	20–24	25–29	30–34	35–39	40–44	45–49	50–54	55–59	60–64	65–69	70–74	75–79	80–84	85–89	> 89
Hypnotics																			
2015	8.0	3.5	3.6	4.4	11.2	23.1	34.7	47.1	58.0	70.6	79.2	79.1	78.7	93.7	140.7	179.3	214.3	237.3	213.9
2016	11.8	11.2	10.1	8.8	13.1	23.1	34.1	45.8	56.8	67.2	79.8	79.7	77.3	91.1	131.6	169.2	204.8	227.3	207.8
2017	7.8	3.7	3.4	4.6	10.2	21.3	32.8	44.7	55.7	66.5	77.6	80.4	77.1	87.3	117.9	159.8	189.6	209.0	196.2
2018	12.0	11.2	10.4	10.5	13.2	23.2	34.2	45.3	56.9	67.1	79.0	84.3	80.2	89.5	116.6	162.0	196.3	216.3	208.7
Anxiolytics																			
2015	0.0	0.2	1.0	3.7	11.3	23.9	36.7	50.8	60.2	68.2	69.9	65.0	54.5	53.7	65.6	79.7	93.1	97.5	83.4
2016	0.0	0.2	1.0	3.5	10.3	22.4	34.6	48.5	58.4	64.6	69.3	64.2	53.1	51.4	60.0	72.0	85.0	90.3	78.4
2017	0.0	0.2	0.9	3.4	9.7	21.0	32.7	46.0	56.3	62.6	66.4	62.9	52.0	48.4	53.5	65.2	75.7	80.9	71.7
2018	0.0	0.2	0.9	3.5	9.3	19.7	31.6	43.9	54.8	60.8	65.1	63.4	52.4	47.5	51.1	60.6	71.4	76.1	68.4
BZRAs																			
2015	0.0	0.1	1.1	5.6	20.2	44.7	68.9	95.3	115.4	135.8	145.9	140.7	129.8	143.4	200.1	250.3	295.6	320.2	281.7
2016	0.0	0.1	1.1	5.4	18.3	41.6	65.1	90.8	111.6	128.1	144.7	139.6	126.0	137.4	184.0	230.6	275.2	298.5	265.6
2017	0.0	0.1	1.0	5.1	17.4	39.4	62.3	87.0	108.2	124.8	139.3	138.4	124.4	130.8	165.1	215.9	253.1	274.4	250.3
2018	0.0	0.1	1.0	5.2	17.0	37.8	61.2	84.2	106.8	122.5	138.1	141.2	126.2	129.4	157.5	207.1	246.2	264.0	243.8
Z-drugs																			
2015	0.0	0.0	0.1	0.6	2.1	4.5	6.5	8.6	10.2	12.7	15.0	16.1	17.0	22.1	36.8	48.3	59.3	66.5	60.6
2016	0.0	0.0	0.1	0.6	2.1	4.5	6.6	8.7	10.2	12.4	15.4	16.6	16.9	21.6	34.2	45.7	56.3	63.2	58.3
2017	0.0	0.0	0.1	0.6	2.2	4.8	7.1	9.1	10.8	13.0	16.0	17.5	17.9	21.9	32.1	46.0	55.4	62.3	59.0
2018	0.0	0.0	0.1	0.7	2.3	5.0	7.5	9.4	11.5	13.5	16.5	18.6	18.9	22.4	31.3	45.7	55.7	61.5	58.8

*BZRAs* benzodiazepine receptor agonists

Table 3 shows the prescription amount (Diazepam-equivalent doses in milligrams) per capita by type of drug for women. BZRAs comprise most of the amount of prescriptions of hypnotics and anxiolytics as in men. The amount of prescriptions of hypnotics and anxiolytics tended to be greater compared to men in older ages, and the decrease in Z-drugs prescriptions for women over the years was not observed in the oldest age groups, unlike that which was observed in men.

**Table 3:** Prescription amount (Diazepam-equivalent doses in milligrams) per capita by type of drugs for women

	Age group																		
Year	0–4	5–9	10–14	15–19	20–24	25–29	30–34	35–39	40–44	45–49	50–54	55–59	60–64	65–69	70–74	75–79	80–84	85–89	> 89
Hypnotics																			
2015	6.7	2.9	2.5	6.2	19.0	35.0	44.4	55.5	66.0	74.2	81.8	86.1	93.4	116.9	178.1	229.5	269.0	265.9	211.1
2016	10.4	8.1	7.8	9.5	20.1	35.0	43.9	53.9	64.9	71.4	81.9	85.5	90.1	112.8	167.1	216.2	259.1	260.3	209.2
2017	6.4	3.1	2.9	6.0	17.2	33.0	42.8	52.6	63.8	71.2	79.5	85.2	89.1	107.5	150.4	204.1	242.6	246.0	199.8
2018	10.9	9.3	8.7	10.5	20.1	35.6	44.6	53.9	65.3	73.3	81.7	89.0	91.8	108.7	148.2	205.4	250.0	259.0	214.9
Anxiolytics																			
2015	0.0	0.2	1.2	6.1	19.4	35.9	48.0	60.9	72.3	80.3	85.2	84.4	84.9	97.6	128.2	157.6	175.3	159.6	111.2
2016	0.0	0.1	1.1	5.8	17.8	33.8	45.9	58.7	70.2	76.8	84.1	82.7	80.7	92.6	118.1	142.5	162.6	151.5	107.1
2017	0.0	0.1	1.1	5.8	16.9	31.9	44.2	56.1	67.7	74.7	79.2	79.0	76.4	85.3	104.4	128.4	146.0	138.6	100.4
2018	0.0	0.2	1.1	5.9	16.4	30.8	42.9	54.8	66.3	73.4	78.2	79.0	75.1	81.5	98.3	118.8	137.8	132.8	97.1
BZRAs																			
2015	0.0	0.1	1.3	9.7	35.7	68.3	89.8	113.4	134.9	150.0	162.0	165.3	173.1	208.3	297.1	374.5	428.7	409.0	307.3
2016	0.0	0.1	1.3	9.2	32.6	64.0	86.0	108.6	130.7	143.0	160.0	162.2	164.8	198.1	274.9	344.5	403.2	391.1	296.8
2017	0.0	0.1	1.2	9.1	31.1	61.2	83.2	104.4	126.7	140.5	152.4	157.8	159.4	186.3	246.4	320.8	373.4	367.7	284.0
2018	0.0	0.1	1.2	9.4	30.4	60.3	81.9	103.1	125.3	140.0	152.2	159.7	158.6	180.6	233.3	304.6	360.9	359.9	279.9
Z-drugs																			
2015	0.0	0.0	0.1	1.0	3.9	7.3	9.7	12.0	13.7	15.4	18.1	20.9	24.2	31.9	51.6	67.4	78.6	76.7	59.2
2016	0.0	0.0	0.1	1.0	3.7	7.3	9.7	12.0	14.0	15.4	18.6	21.1	23.8	31.3	49.0	64.3	76.5	75.4	58.7
2017	0.0	0.0	0.1	1.1	3.9	7.8	10.2	12.5	14.7	16.3	19.0	22.0	24.7	31.7	46.8	64.9	76.7	76.4	60.8
2018	0.0	0.0	0.1	1.3	4.2	8.5	10.7	13.2	15.5	17.1	19.9	23.2	25.6	32.0	45.6	64.4	77.2	77.8	61.9

*BZRAs* benzodiazepine receptor agonists

Table 4 shows the SCRs of hypnotics and anxiolytics for each prefecture and year. The SCR of hypnotics for Hokkaido was the largest every year, and the SCR of anxiolytics for Akita was the largest every year. On the other hand, the SCR for Fukui tended to be the smallest among prefectures both for hypnotics and anxiolytics.

**Table 4:** The SCRs of hypnotics and anxiolytics for each prefecture and year

	Hypnotics				Anxiolytics			
Prefecture	2015	2016	2017	2018	2015	2016	2017	2018
Hokkaido	132.7	131.6	130.9	132.3	123.0	125.3	123.5	126.8
Aomori	81.5	83.0	82.3	82.9	127.4	128.7	124.7	124.3
Iwate	86.3	85.1	86.3	86.3	118.8	118.5	116.0	117.4
Miyagi	99.8	99.7	96.8	97.3	122.0	121.5	120.2	120.8
Akita	111.0	110.1	110.5	110.1	151.1	150.1	148.2	145.8
Yamagata	106.2	107.3	109.1	108.5	111.1	112.0	111.0	112.0
Fukushima	113.9	112.6	113.4	112.7	120.5	121.6	122.1	121.3
Ibaraki	82.0	83.0	83.2	82.4	92.4	91.4	90.8	91.3
Tochigi	93.8	93.7	92.7	92.7	103.8	102.4	101.2	100.8
Gunma	100.9	102.1	102.7	102.9	108.4	109.0	109.7	111.7
Saitama	81.2	81.4	81.3	80.8	89.9	90.5	90.9	90.3
Chiba	80.7	82.1	80.9	82.2	87.7	87.6	87.6	87.5
Tokyo	112.0	112.3	111.0	110.1	113.5	113.5	114.1	113.2
Kanagawa	88.6	88.6	87.5	89.0	89.9	91.2	91.9	91.4
Niigata	94.7	93.3	93.1	92.3	111.9	110.0	107.8	106.0
Toyama	84.9	83.6	85.9	86.2	89.7	89.4	87.9	90.3
Ishikawa	85.8	84.6	85.8	85.9	89.0	88.1	87.3	86.6
Fukui	84.1	84.1	85.7	84.1	80.0	79.9	78.8	79.2
Yamanashi	97.8	95.7	92.5	90.7	87.9	85.8	83.5	83.1
Nagano	101.4	102.9	102.5	102.6	98.3	98.3	98.6	98.9
Gifu	94.2	94.3	94.8	94.9	88.1	87.5	87.9	87.8
Shizuoka	87.1	88.3	88.1	88.3	82.4	83.0	83.3	84.8
Aichi	99.4	100.4	99.4	100.7	93.6	92.5	91.5	90.5
Mie	89.8	89.7	91.8	90.3	105.9	104.5	104.8	102.7
Shiga	90.9	87.0	87.9	88.7	86.3	83.8	83.6	82.0
Kyoto	105.8	104.5	105.1	104.0	84.9	83.4	82.7	82.3
Osaka	110.4	110.9	110.5	110.4	98.9	98.7	99.8	100.0
Hyogo	100.6	99.2	98.7	98.0	99.7	99.3	99.1	98.8
Nara	90.9	91.5	93.2	92.9	90.2	90.2	91.0	91.6
Wakayama	113.2	114.6	118.5	117.1	121.3	122.3	123.6	124.3
Tottori	87.0	85.3	85.2	82.9	87.8	89.0	88.7	88.1
Shimane	107.6	107.3	107.4	106.2	96.9	96.5	98.1	97.9
Okayama	110.1	108.3	107.3	104.2	97.2	96.2	96.1	94.8
Hiroshima	113.1	112.1	110.6	109.8	101.3	99.9	100.2	99.9
Yamaguchi	94.0	94.1	92.3	93.7	101.6	101.5	102.4	103.6
Tokushima	90.2	89.9	90.8	90.4	100.2	100.5	100.9	102.0
Kagawa	97.1	97.6	97.7	100.1	97.8	97.7	98.0	98.4
Ehime	100.5	101.5	103.7	104.5	99.0	97.7	98.6	100.3
Kochi	101.3	102.9	99.8	100.9	109.8	109.1	108.9	108.2
Fukuoka	116.2	116.4	119.8	117.8	91.9	93.2	94.0	94.7
Saga	101.6	103.0	107.2	105.8	84.4	84.4	86.1	87.0
Nagasaki	108.5	106.9	111.3	112.2	92.7	93.7	94.9	95.7
Kumamoto	90.6	92.1	93.4	94.8	98.1	101.1	101.3	101.4
Oita	113.3	111.2	117.2	116.1	98.8	99.5	101.0	102.2
Miyazaki	106.0	106.9	107.4	107.8	98.4	98.0	97.2	98.1
Kagoshima	90.4	90.7	92.9	95.5	97.5	96.5	98.9	99.8
Okinawa	95.5	94.7	98.3	100.0	95.3	95.8	95.9	95.5

*SCR* standardized claim ratio

Table 5 shows the SCRs of BZRAs and Z-drugs for each prefecture and year. The SCR for Hokkaido and Akita were particularly large in the periods for BZRAs. On the other hand, the SCR for Nagasaki was the largest for Z-drugs throughtout the periods.

**Table 5:** The SCRs of BZRAs and Z-drugs for each prefecture and year

	BZRAs				Z-drugs			
Prefecture	2015	2016	2017	2018	2015	2016	2017	2018
Hokkaido	128.7	129.1	128.1	130.8	122.4	117.4	116.3	117.5
Aomori	100.4	101.3	99.1	98.4	77.8	78.4	78.2	77.8
Iwate	100.0	99.1	98.5	98.6	88.2	84.4	84.1	84.8
Miyagi	109.9	109.0	107.1	106.9	97.0	96.5	92.8	91.1
Akita	128.4	127.3	126.6	124.7	103.2	99.5	100.0	100.5
Yamagata	108.3	109.5	110.2	110.1	92.1	91.9	93.9	94.1
Fukushima	117.2	117.6	117.7	117.3	115.5	114.2	113.2	112.6
Ibaraki	86.3	86.6	86.2	86.1	67.9	69.4	70.4	72.3
Tochigi	98.2	97.8	96.3	96.4	84.3	83.7	82.2	84.3
Gunma	103.7	105.3	105.7	107.3	73.7	74.8	76.2	79.0
Saitama	85.2	85.8	85.7	85.3	78.3	81.7	81.9	83.2
Chiba	83.6	84.1	83.5	84.1	82.9	83.1	83.1	82.0
Tokyo	112.9	113.3	112.6	112.2	118.0	121.6	122.0	123.0
Kanagawa	89.2	89.6	89.6	89.9	92.3	94.8	96.1	97.0
Niigata	101.9	100.1	99.0	97.4	95.5	93.3	91.1	89.9
Toyama	86.9	86.1	86.2	87.8	84.5	80.9	79.5	81.8
Ishikawa	86.8	85.7	85.3	85.0	105.8	103.5	102.2	101.8
Fukui	82.0	81.8	82.1	81.0	95.1	91.6	90.6	91.7
Yamanashi	92.6	90.5	87.9	85.9	103.4	102.4	99.0	96.3
Nagano	99.8	100.2	100.4	99.5	83.1	84.7	87.0	87.4
Gifu	92.3	92.6	92.9	93.3	103.9	103.4	102.3	101.7
Shizuoka	84.6	85.0	85.5	84.8	90.1	90.8	90.9	90.5
Aichi	96.8	96.6	95.8	95.9	102.5	102.1	100.0	99.4
Mie	95.8	95.7	96.5	95.6	78.5	80.5	80.9	81.6
Shiga	87.9	84.7	85.1	85.2	80.9	79.4	80.9	82.9
Kyoto	97.1	96.3	96.2	96.4	98.4	97.5	95.8	95.8
Osaka	105.5	105.4	106.1	106.3	112.1	111.5	111.8	111.5
Hyogo	100.0	99.1	98.7	98.4	109.9	108.1	106.1	105.6
Nara	90.3	90.8	91.9	92.5	91.2	90.7	89.8	89.3
Wakayama	116.6	118.1	121.1	121.6	102.7	104.5	106.1	102.9
Tottori	87.9	87.8	87.0	84.8	80.3	77.3	75.6	74.4
Shimane	103.9	104.3	104.2	103.6	112.8	109.7	110.8	112.0
Okayama	103.7	102.7	101.3	99.5	98.6	93.9	93.4	92.3
Hiroshima	108.0	106.4	106.0	104.5	108.7	106.3	105.2	102.4
Yamaguchi	97.3	97.1	96.3	97.0	117.3	113.4	113.4	111.9
Tokushima	94.6	94.9	95.1	95.3	95.0	96.0	93.6	90.7
Kagawa	97.4	97.2	98.2	98.7	86.8	86.3	85.4	86.4
Ehime	99.8	99.8	101.6	102.7	103.5	104.1	103.7	103.9
Kochi	105.0	104.8	103.7	102.7	115.8	114.3	112.8	110.7
Fukuoka	106.2	107.3	109.4	109.1	107.2	107.4	108.8	106.5
Saga	94.5	95.7	98.6	98.7	98.1	99.6	98.7	95.7
Nagasaki	101.2	101.2	104.2	105.8	134.5	131.3	133.0	133.4
Kumamoto	92.8	93.6	94.6	94.2	103.7	103.9	106.4	107.9
Oita	106.6	106.7	110.7	112.1	104.4	104.4	107.6	107.6
Miyazaki	103.3	103.1	103.9	104.1	114.5	115.0	116.7	115.8
Kagoshima	94.0	93.4	95.7	98.0	96.5	100.2	105.7	107.8
Okinawa	95.4	94.5	97.1	96.0	108.2	106.0	111.6	109.6

*SCR* standardized claim ratio; *BZRAs* benzodiazepine receptor agonists

Table 6 shows the results of regression analysis. The number of medical clinics per 100,000 persons, the number of public assistance recipients per 1000 persons, the proportion of persons whose HbA1c ≥ 6.5%, and the proportion of persons whose BMI ≥25 kg/m^2^ were positively and significantly associated with the SCR of hypnosis. In contrast, The number of public assistance recipients per 1000 persons and the proportion of persons whose BMI ≥25 kg/m^2^ were positively and significantly associated with the SCR of anxiolytics.

**Table 6:** Result of the regression analysis

	Hypnotics		Anxiolytics	
Explanatory variable	SPRC (95% CI)	*p*-value	SPRC (95% CI)	p-value
Year	−0.096 (− 0.202, 0.008)	0.081	− 0.055 (− 0.132, 0.020)	0.161
Medical characteristics				
Number of hospitals per 100,000 persons	0.141 (−0.267, 0.540)	0.506	−0.215 (− 0.551, 0.123)	0.205
Number of medical clinics per 100,000 persons	0.296 (0.049, 0.551)	0.026	0.001 (−0.206, 0.210)	0.996
Number of pharmacies per 100,000 persons	0.152 (−0.034, 0.343)	0.122	−0.004 (− 0.140, 0.137)	0.960
Number of hospitals with a psychiatric department per 100,000 persons	0.018 (−0.178, 0.219)	0.858	0.051 (−0.079, 0.185)	0.453
Number of psychiatric beds per 100,000 persons	−0.347 (− 0.751, 0.064)	0.105	0.148 (−0.155, 0.451)	0.350
Socioeconomic characteristics				
Number of public assistance recipients per 1000 persons	0.296 (0.072, 0.522)	0.015	0.284 (0.096, 0.476)	0.004
Taxable income per capita	−0.138 (− 0.403, 0.131)	0.326	−0.075 (− 0.280, 0.131)	0.486
Physical characteristics				
Proportion of persons whose HbA1c ≥ 6.5%	0.142 (0.078, 0.205)	< 0.001	0.029 (−0.011, 0.068)	0.165
Proportion of persons whose SBP ≥ 140 mmHg	−0.045 (− 0.122, 0.032)	0.230	0.024 (−0.024, 0.073)	0.327
Proportion of persons whose BMI ≥ 25 kg/m^2^	0.289 (0.031, 0.545)	0.032	0.201 (0.008, 0.398)	0.045
Proportion of persons whose triglycerides ≥150 mg/dl	−0.100 (− 0.204, 0.009)	0.074	−0.034 (− 0.103, 0.037)	0.338

*SPRC* standardized partial regression coefficient; *CI* confidence intervals; *SBP*, systolic blood pressure; *BMI* body mass index

## Discussion

We showed trends and regional differences in the amount of prescriptions of hypnotics and anxiolytics in all of Japan in recent years. We also identified factors associated with regional differences. BZRAs account for most of the prescriptions of hypnotics and anxiolytics in Japan, whereas the amount of BZRAs prescriptions decreased in the periods. We interpret the results and discuss possible reasons for the results below.

One possible factor for the decrease of BZRA prescriptions is the recent publication of guidelines concerning these prescriptions. The Japan Geriatrics Society published the “Guidelines for Medical Treatment and Its Safety In The Elderly 2015.” In this guideline, benzodiazepines and Z-drugs (non-benzodiazepines) were designated drugs to be prescribed with caution in elderly persons [20]. BZRAs consist of benzodiazepines and Z-drugs [21, 22]. Benzodiazepines were already not recommended in some guidelines before the analysis periods [23, 24], and Z-drugs were also designated as drugs that need to be prescribed with caution in 2015. BZRAs comprise most of the prescribed hypnotics and anxiolytics in Japan, and a decrease in the amount of BZRAs prescriptions led to a decrease in hypnotics and anxiolytics. In addition, some medical fee revisions were conducted in the 2010s to regulate multidrug prescriptions of hypnotics and anxiolytics, as explained in the Introduction [1], and it is also considered related to the decrease of amount of prescriptions.

Number of medical clinics per capita was found to be associated with regional differences in the prescription amounts of hypnotics. It might be natural that the prescription amount increases as the number of medical clinics with a psychiatric department increase because psychotropic drugs are prescribed in medical clinics; however, it is unclear why the association was observed only for hypnotics.

The number of public assistance recipients was positively associated with the regional amount of hypnotics and anxiolytics prescriptions. There is little evidence for an association between socioeconomic indicators and use of hypnotics or anxiolytics in Japan [7]. However, the prevalence of psychological distress is higher in persons with a low socioeconomic status in Japan [25, 26]. In addition, there is an association between unemployment and insomnia symptoms among working-age persons in Japan [27]. One study showed that higher-grade employees had better sleep quality than lower grades in Japanese men [28]. Moreover, patients with sleep disturbances in whom hypnotics and anxiolytics are prescribed have some difficulties in working than the general population [29, 30], and some of these patients may become public assistance recipients. The prevalence of psychiatric diseases among public assistance recipients is known to be high in Japan [31]. Therefore, it is considered that the high prevalence of sleep disorder and anxiety symptoms in persons of low socioeconomic status or patients with psychiatric diseases in a prefecture affected many anxiolytics and hypnotics prescriptions in the prefecture.

Prevalence of obesity was positively associated with a higher amount of the prescriptions of hypnotics and anxiolytics, and prevalence of diabetes was also positively associated with the prescription of hypnotics. Obesity was correlated with high psychiatric agent use among prefectures in Japan [8], and this study indicated a similar result for hypnotics and anxiolytics. Sleep disturbance and obesity are closely associated, and sleep disturbance and obesity mutually affect each other [32]. In addition, sleeping pill users had a higher risk of metabolic syndrome in Japan [33]. Moreover, an association between psychiatric disease and diabetes [34] is known, and a high prevalence of obesity and diabetes was observed in outpatients with schizophrenia in Japan [35]. Mental health problems are comorbidities of diabetes [36], and diabetes or obesity patients often use hypnotics and anxiolytics. In addition, it is considered that patients with psychiatric diseases tend to be obese or diabetic because occurring psychiatric diseases can lead to poverty. Low socioeconomic status is associated with a high prevalence of obesity or diabetes in Japan [37, 38].

No studies investigated regional differences in sleep disorders or anxiety disorders in Japan, and it is considered that regional differences in the amount of hypnotics and anxiolytics prescriptions indicate differences in the number of patients with these diseases. However, BZRAs comprise most hypnotics and anxiolytics prescriptions in Japan, and patients in prefectures with more prescriptions are more exposed to BZRAs. Therefore, prefectures with a particularly high amount of the prescriptions need to take measures to reduce them for the health of residents. Prefectures with a particularly high amount of the prescriptions need to protect high-risk persons, such as individuals with low socioeconomic status and patients with lifestyle-related diseases, from occuirng psychiatric diseases. In addition, there might be cases where unnecessary multidrug prescriptions of hypnotics and anxiolytics are habitually provided, and it is important for physicians in regions with a particularly high amount of the prescriptions to recheck their prescriptions behaviors. Moreover, it will be meaningful to investigate the current conditions of prescriptions against public assistance recipients to determine whether they received inappropriate multidrug prescriptions. Furthermore, differences in the amount of hypnotics and anxiolytics prescriptions affect regional differences in medical costs. The social cost of insomnia and anxiety disorder is estimated to be enormous in Japan [39, 40]. Therefore, finding a way to reduce the regional difference in the amount of prescriptions will help reduce medical costs in Japan.

There are several limitations to this study. First, it is an ecological study, and an epidemiological study investigating an association between the amount of prescriptions and persons’ physical and socioeconomic characteristics using individual data is needed to verify the results. Second, there were a few drugs whose titer (force value) was unknown, and we complemented the mean of titers for these drugs. A study investigating titers of new drugs is also warranted. Third, data on the top 100 drugs whose prescribed number is high are publicly available for each therapeutic category in the NDB Open data, and data on drugs whose prescribed number is low are not available. A study using NDB data comprising all hypnotics and anxiolytics is also needed to verify the results of this study. Similarly, there were cases where the prescribed amount of some drugs was unavailable in some age groups because the prescribed amount is not published in the NDB Open data when the prescribed number is below 10 in an age group. Fourth, it is also important to know the number of patients prescribed with hypnotics and anxiolytics or the prescribed amount per patient, whereas these values are unavailable from the NDB Open data. A study investigating these indicators using NDB data is also warranted. Additionally, there is a possibility that a decrease in the prescription amount was caused by the decrease in the number of patients; however, the prevalence of mental service use has increased in the past decade in Japan [41]. Moreover, the estimated number of patients with neurotic, stress-related, and somatoform disorders, psychiatric diseases, and sleep disorders has been increasing in Japan over recent decades [42]. Therefore, it is likely that the decrease in the prescription is not caused by the decrease in the number of patients or medical service use rates. Fifth, the diagnosis and indication might have changed over the analyzed years, and it is a possible confounder of the trend observed in prescription amounts. However, there is no evidence of change in the diagnosis criteria or decrease in indication of the psychotropic drugs over the analyzed time periods. A study using individual-level data is warranted to take into account for disease prevalence in the analysis.

Sixth, we used the data of Special Health Checkups, which cover only physical characteristics of persons aged 40–74 years. We used this data because they are national data covering approximately half of all persons aged 40–74 years in Japan and because it is the only available source of national data that covers the physical characteristics of each prefecture in Japan over the analyzed time periods. On the other hand, data of the physical characteristics do not represent the true values of persons in all ages for prefectures, and statistically biased estimates were obtained in this study. However, we could not evaluate degree of the bias for using these data, and this is a limitation of this study. It is also possible that the results do not much change when using true values of physical characteristics instead of the Specific Health Checkups data because it is considered that the values of physical characteristics for persons aged 40–74 years and those of all ages are correlated among prefectures. Actually, it was shown in previous studies that aggregated values of the physical characteristics in the Specific Health Checkups were significantly associated with population health outcomes (i.e., life expectancy and healthy life expectancy) across prefectures [43, 44].

## Conclusions

We investigated the regional differences in the amount of hypnotics and anxiolytics prescriptions in Japan and identified factors associated with these regional differences by an ecological study. The amount of hypnotics and anxiolytics prescriptions, specifically those for BZRAs, decreased in many age groups in adults from 2015 to 2018. The regression analysis revealed that the number of medical clinics per capita, the number of public assistance recipients per capita, the proportion of persons whose HbA1c ≥ 6.5%, and the proportion of persons whose BMI ≥25 kg/m^2^ were positively and significantly associated with the SCR of hypnosis. In contrast, The number of public assistance recipients per capita and the proportion of persons whose BMI ≥25 kg/m^2^ were positively and significantly associated with the SCR of anxiolytics.

## Data Availability

The data used in this study can be obtained from websites of government statistics in Japan. Population data were obtained from the Survey of the basic resident register ([cited 1 August 2021]. Available from: https://www.e-stat.go.jp/stat-search/files?page=1&toukei=00200241&tstat=000001039591). Data on the medical and socioeconomic characteristics in prefectures were obtained from e-Stat General counter for government statistics([cited 1 August 2021]. Available from: https://www.e-stat.go.jp/regional-statistics/ssdsview). Data on the prescription amount and the physical characteristics for prefectures were obtained from NDB Open data ([cited 1 August 2021]. Available from: https://www.mhlw.go.jp/stf/seisakunitsuite/bunya/0000177182.html).

## Abbreviations

SPRC: standardized partial regression coefficient
CI: confidence intervals
SBP: systolic blood pressure
BMI: body mass index
SCR: standardized claim ratio
BZRAs: Benzodiazepine receptor agonists
NDB: National Database of Health Insurance Claims and Specific Health Checkups of Japan
